# Knockdown of TANK-Binding Kinase 1 Enhances the Sensitivity of Hepatocellular Carcinoma Cells to Molecular-Targeted Drugs

**DOI:** 10.3389/fphar.2022.924523

**Published:** 2022-06-07

**Authors:** Fengxia Du, Huiwei Sun, Fang Sun, Shiwei Yang, Haidong Tan, Xiaojuan Li, Yantao Chai, Qiyu Jiang, Dongdong Han

**Affiliations:** ^1^ Department of Pharmacy, Medical Support Center of PLA General Hospital, Beijing, China; ^2^ Department of Infectious Diseases, Fifth Medical Center of Chinese PLA General Hospital, Institute of Infectious Diseases, Beijing, China; ^3^ Organ Transplant Center and Department of Hepatobiliary Surgery, China-Japan Friendship Hospital, Beijing, China

**Keywords:** TANK-binding kinase 1, advanced hepatocellular carcinoma, molecular-targeted drugs, epithelialmesenchymal transition, drug resistance, pro-survival, anti-apoptosis

## Abstract

The protein kinase, TANK-binding kinase 1 (TBK1), not only regulates various biological processes but also functions as an important regulator of human oncogenesis. However, the detailed function and molecular mechanisms of TBK1 in hepatocellular carcinoma (HCC), especially the resistance of HCC cells to molecular-targeted drugs, are almost unknown. In the present work, the role of TBK1 in regulating the sensitivity of HCC cells to molecular-targeted drugs was measured by multiple assays. The high expression of TBK1 was identified in HCC clinical specimens compared with paired non-tumor tissues. The high level of TBK1 in advanced HCC was associated with a poor prognosis in patients with advanced HCC who received the molecular-targeted drug, sorafenib, compared to patients with advanced HCC patients and a low level of TBK1. Overexpression of TBK1 in HCC cells induced their resistance to molecular-targeted drugs, whereas knockdown of TBK1 enhanced the cells’ sensitivity to molecular-targeted dugs. Regarding the mechanism, although overexpression of TBK1 enhanced expression levels of drug-resistance and pro-survival-/anti-apoptosis-related factors, knockdown of TBK1 repressed the expression of these factors in HCC cells. Therefore, TBK1 is a promising therapeutic target for HCC treatment and knockdown of TBK1 enhanced sensitivity of HCC cells to molecular-targeted drugs.

## Introduction

Hepatocellular carcinoma (HCC) is the most common and frequent primary liver tumor, and its risk factors are mainly viral hepatitis and alcohol abuse (Lin et al., 2020; Nhlane et al., 2021; Powell et al., 2021). Despite the progress made in neonatal Hepatitis B virus (HBV) vaccination and HBV anti-tumor symptomatic treatment, there are still more than 80 million people infected with HBV and other hepatitis viruses (HCV) in China (Polaris Observatory Collaborators, 2018; Sung et al., 2021; Polaris Observatory HCV Collaborators, 2022). Due to the high HBV and HCV infection rates in East Asia and the Asia-Pacific region represented by China, and as the diseases progress, patients have a high risk of eventually developing HCC (Wang et al., 2014; Zhang S et al., 2017; Tan and Schreiber, 2020). HCC has an insidious onset and a long disease course (Wang et al., 2014; Zhang S et al., 2017; Tan and Schreiber, 2020). Most patients are in an advanced stage at the first diagnosis and cannot receive radical treatment strategies such as liver transplantation or surgical resection (Kim et al., 2017; Llovet et al., 2018; Wang and Wei, 2020). For advanced HCC, one of the main drug treatment strategies is molecular-targeted therapy: patients orally take various small-molecule, multi-target protein kinase inhibitors (Roskoski, 2019; Roskoski, 2020; Roskoski, 2021; Roskoski, 2022). Although global, multi-center clinical trials show that these molecular-targeted drugs can help delay disease progression in patients with HCC, improve patients’ quality of life, and prolong patients’ survival (Bruix et al., 2017; Kudo et al., 2018), these drugs still have many shortcomings: 1) individual differences in patients with advanced HCC on molecular-targeted drugs treatment are very large, and only some patients are sensitive to molecular-targeted drugs (Zhu et al., 2017; Tang et al., 2020); 2) the treatment cycle of molecular-targeted drugs is very long, and patients are prone to develop drug resistance as treatment progresses (Zhu et al., 2017); and 3) these drugs are toxic and side effects of molecular-targeted drugs cannot be ignored (Zhu et al., 2017). Although many advances have been made in research on molecular-targeted drugs, the molecular mechanisms leading to drug resistance in HCC is still unclear and there is no ideal indicator molecule to signal the prognosis of patients receiving molecular-targeted drug therapy (Zhu et al., 2017). Therefore, there is a great need to elucidate the molecular mechanism of HCC resistance to molecular-targeted drugs and to study and discover new intervention targets to achieve safer and more effective molecular-targeted therapy.

As a member of the ubiquitous serine/threonine kinases that play important roles in regulating immune or inflammatory responses, the TRAF-associated NF-κB activator (TANK) binding kinase 1 (TBK1) has been considered as a therapeutic target (Li et al., 2017; Xu et al., 2020; Taft et al., 2021). It was initially considered an activator of the NF-κB pathway *via* inhibiting the activation of the IKK [inhibitor of nuclear factor-κB (IκB) kinase]-related pathway (Alam et al., 2021). Recently, aberrant TBK1 expression and/or activity have been identified in various human malignancies, including lung cancer, pancreatic cancer, breast cancer, and colorectal cancer (Revach et al., 2020; Alam et al., 2021; Herhaus, 2021). TBK1 has also been considered to be an oncogene (Revach et al., 2020; Alam et al., 2021; Herhaus, 2021). However, the roles of TBK1 in HCC are still unclear. Moreover, inflammation is closely related to the occurrence and progression of tumors (Donisi et al., 2020; Heinrich et al., 2021; Huang et al., 2021). NF-κB and its related signaling pathways not only play an important role in the body’s immune response and physiological mechanisms such as inflammation but also promote the occurrence and progression of various malignant tumors (Yang et al., 2019; Jiang et al., 2021a; Zhou Q et al., 2021). In addition, NF-κB can also induce the resistance of malignant tumor cells to anti-tumor drugs (Ding et al., 2021; Kumar et al., 2021; Shen et al., 2021; Smith and Burger, 2021). To this end, the present study intends to systematically investigate the molecular mechanism by which TBK1 regulates the resistance of HCC cells to molecular-targeted drugs. Exploring the significance of TBK1 as an intervention target sensitize HCC cells to molecularly-targeted drugs is of great value.

## Materials and Methods

### Cell Lines and Vectors

The cell lines used in this study were mainly liver-derived, non-tumor cell lines (L-02) and some HCC cell lines (including MHCC97-H, MHCC97-L, HepG2, Huh-7, BEL-7402, and SMMC-7721). These cell lines are maintained in our laboratory and detailed in previous publications (He X et al., 2021; Yang H et al., 2021; Jiang Q et al., 2021; Li et al., 2021). The expression levels assessed in these experiments included the full-length sequence of *TBK1* and its siRNA, which were prepared as the lentivirus (these were transduced into lentivirus vectors). The target sequence of *TBK1* siRNA was 5′-TAA​ACT​TCT​ATT​AGA​AAG​CTA-3′ and *siTBK1* was used in pcilencer2.1U6 vectors. All sequences were confirmed by DNA sequencing. The cells were infected with the viral vectors and cells with the neomycin-resistance selectable marker were screened and selected by treatment with G418.

### Clinical Specimens and qPCR

Clinical tissue specimens were obtained from patients with advanced HCC (52 patients) and paired, non-tumor tissues from the same patients were also obtained. These specimens were maintained in our laboratory and used as detailed in previous publications (Feng et al., 2018; Shao et al., 2018; Zhang et al., 2018). The expression levels of *TBK1* and other factors were examined by qPCR according to previous publications and manufacturer instructions. The primers used in these experiments were: E-cadherin, 5′-CTC​CTG​AAA​AGA​GAG​TGG​AAG​TGT-3′; 5′-CCG​GAT​TAA​TCT​CCA​GCC​AGT​T-3′; N-cadherin, 5′-CCT​GGA​TCG​CGA​GCA​GAT​A-3′; 5′-CCA​TTC​CAA​ACC​TGG​TGT​AAG​AAC-3′; vimentin, 5′-ACC​GCA​CAC​AGC​AAG​GCG​AT-3′; 5′-CGA​TTG​AGG​GCT​CCT​AGC​GGT​T-3′; BCL2, 5′-GAT​CGT​TGC​CTT​ATG​CAT​TTG​TTT​TG-3′; 5′-CGG​ATC​TTT​ATT​TCA​TGA​GGC​AC GTTA-3′; NICD (Notch NICD, the intracellular domain of Notch protein), 5′-CCGACGCACA AGGTGTCTT-3′, 5′-GTC​GGC​GTG​TGA​GTT​GAT​GA-3′; survivin, 5′-ACATGCAGCTCGAATG AGAACAT-3′, 5′-GAT​TCC​CAA​CAC​CTC​AAG​CCA-3′; cIAP-1, 5′-GTG​TTC​TAG​TTA​ATC​CTG AGCAGCTT-3′; 5′-TGG​AAA​CCA​CTT​GGC​ATG​TTG​A-3′; cIAP-2, 5′-CAAGGACCACCG CATCTCT-3′; 5′-AGC​TCC​TTG​AAG​CAG​AAG​AAA​CA-3′; TBK1, 5′-CCCTTTGAAGGGC CTCGTAG-3′; 5′-ACC​CCG​AGA​AAG​ACT​GCA​AG-3′; NF-κB p50 (NFKB), 5′-TTTTCG ACTACGCGGTGACA-3′; 5′-TCC​TGC​ACA​GCA​GTG​AGA​TG-3′; NF-κB p65 (RELA), 5′-TGA​ACC​GAA​ACT​CTG​GCA​GCT​G-3′; 5′-CAT​CAG​CTT​GCG​AAA​AGG​AGC​C-3′; and loading control β-actin, 5′-CAC​CAT​TGG​CAA​TGA​GCG​GTT​C-3′; 5′-AGG​TCT​TTG​CGG​ATG​TCC​A CGT-3′. The heat-map of the qPCR results were obtained according to the methods by Zhou et al., 2020 and Yin et al., 2019 (Yin et al., 2019; Zhou W et al., 2021). The results of qPCR are displayed as heat maps, and the heat maps are drawn based on the relative folds of the expression levels of each factor in each group relative to the control group. At this time, the control group itself has no change compared with itself, so the folds of change is 0, the increase is a positive number of folds, and the decrease is a negative number folds. Each heat-map has a colored ribbon as an indication of the rates/folds of changes.

### Cell-Survival Analysis

The following molecularly-targeted drugs used in this study were obtained by Dr. Cao Shuang of Wuhan Engineering University through chemical synthesis: sorafenib, regorafenib, lenvatinib, cabozantinib, and anlotinib. All pure-drug powders with a purity of greater than 99% were used in this study (see Table 1 referring to the purity of drugs via HPLC [High Performance Liquid Chromatography]). For the cytotoxic chemotherapeutics, etoposide (Cat. No. S1225), adriamycin (Cat. No. S1208), paclitaxel (Cat. No. S1150) or gemcitabine (Cat. No. S1714) was purchased from Selleck Corporation, Houston, Texas, United States. The small molecular inhibitor of TBK1 (MRT67307, Cat. No. S6386) was purchased from Selleck Corporation. For cell experiments, pure powders of these drugs were dissolved in organic solvents, such as DMSO (Dimethyl sulfoxide), according to previous publications (Ma et al., 2016; Wang J. H et al., 2021), then diluted in DMEM (Dulbecco’s Modified Eagle Medium) without FBS (fetal bovine serum). The concentration of molecular-targeted drugs used in the cell-survival analysis were: 30 μmol/L, 10 μmol/L, 3 μmol/L, 1 μmol/L, 0.3 μmol/L, 0.1 μmol/L, 0.03 μmol/L, and 0.01 μmol/L. The concentrations of cytotoxic chemotherapeutics were listed in Table 2. The cells were treated with various concentrations of the drugs for 48 h and counts of living cells were determined by the MTT assay. The inhibitory rates, or the IC_50_ values (half rate of inhibition), of the drugs on HCC cell survival were calculated according to previously published methods (Ma et al., 2016; Wang J. H et al., 2021).

**TABLE 1:** The purity of drugs used in the presence work from HPLC.

Drugs	Purity from HPLC (%)
Sorafenib	99.1
Cabozentinib	99.5
Lenvatinib	99.3
Regorafenib	99.2
Anlotinib	99.5

**TABLE 2:** The concentrations of cytotoxic chemotherapies in cell-based assays.

Drugs	Concentrations (μmol/L)						
paclitaxel	0.0003	0.001	0.003	0.01	0.03	0.1	0.3
etoposide	0.003	0.01	0.03	0.1	0.3	1	3
adriamycin	0.001	0.003	0.01	0.03	0.1	0.3	1
gemcitabine	0.001	0.003	0.01	0.03	0.1	0.3	1

### Subcutaneous Tumor Model

First, the sorafenib solution used in the animal experiments was prepared according to the method described in a previous publication (Jia et al., 2016; Feng et al., 2019; Sun et al., 2019). Specifically, pure sorafenib powder was dissolved in PEG400 (polyethylene glycol 400), Tween 80, and DMSO, then diluted with sterilized normal saline (Wang Y et al., 2021; Du et al., 2021; Jie et al., 2021; Zou et al., 2021). The final dose of sorafenib formulation used for treating the nude mice by oral administration was approximately 0.5 mg/kg. The *siTBK1* was transfected in MHCC97-H cells and *TBK1* into MHCC97-L, after which the cells were injected subcutaneously into nude mice. The nude mice were then given sorafenib by oral gavage at doses of 2 mg/kg, 1 mg/kg, 0.5 mg/kg, and 0.2 mg/kg for almost 21 days (once per 2 days). The tumor weights and tumor volumes were examined.

### Statistical Analysis

Statistical analyses were performed by using the SPSS 9.0 statistical software (IBM Corporation, Armonk, NY, United States; two-way ANOVA with the Bonferroni correction). The IC_50_ values of molecular-targeted drugs were calculated by using Origin software (Origin 6.1; OriginLab Corporation, Northampton, MA, United States).

## Results

### TANK-binding kinase 1 Expression is Associated With the Resistance of Sorafenib in Advanced Hepatocellular Carcinoma

**FIGURE 1:**
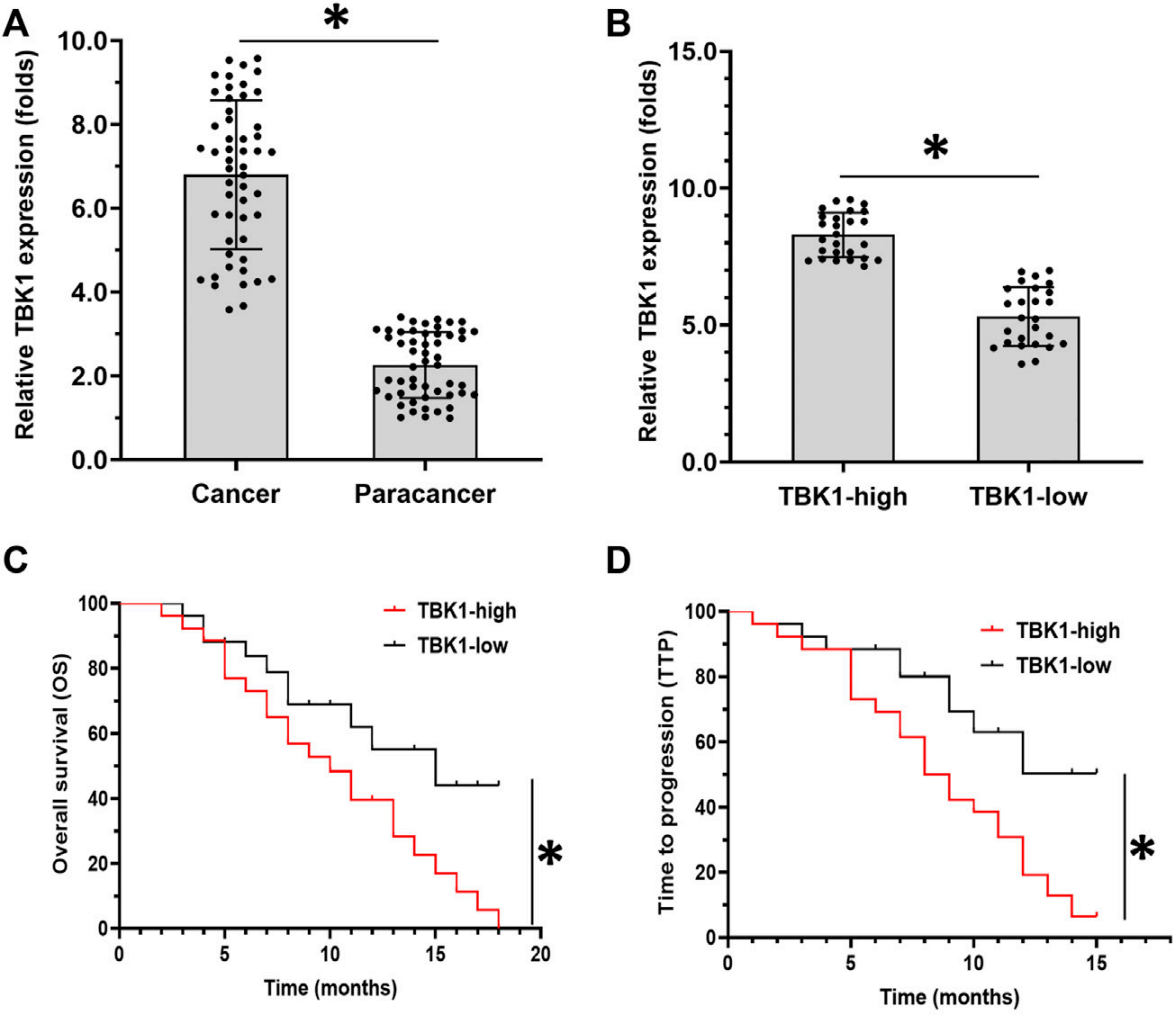
The clinical significance of *TBK1* in HCC. **(A)** The expression of *TBK1* in HCC clinical specimens or paired non-tumor tissues (non-tumor regions of the liver in the HCC patients). **(B)** The median value of *TBK1* in patients in the *TBK1*-high group or the *TBK1*-low group. **(C,** **D)** The OS (overall survival) or TTP (time to progress) of the patients in the *TBK1*-high group or the *TBK1*-low group who received sorafenib treatment. ∗*p* < 0.05.

First, the expression of *TBK1* in clinical specimens was examined. As shown in Figure 1A, the expression levels of *TBK1* were much higher in HCC specimens compared with paired non-tumor tissues. Moreover, the HCC patients were divided into two groups: the *TBK1*-high group or the *TBK1*-low group, according to the median value of the *TBK1* expression levels in the HCC specimens (Figure 1B). The prognosis of patients received sorafneib treatment in the *TBK1*-high group treated with the molecular-targeted drug sorafenib was significantly worse than that of the patients in the *TBK1*-low group. The OS (overall survivial) of the patients in the *TBK1*-high group and the TTP (time to progress) of sorafenib treatment were significantly shorter than those in the *TBK1*-low group (Figures 1C,D and Table 3).

**TABLE 3:** The endogenous TBK1 level associated with the clinical outcome of patients received sorafenib treatment.

	TBK1 mRNA expression		P
	High (*n* = 26)	Low (*n* = 26)	
TTP	8	12	0.01
	6.0–9.9 (M)	9.8–13.4 (M)	
OS	10	15	0.031
	6.6–13.4 (M)	7.7–22.2 (M)	

TTP, time to progress; OS, overall survival.

**FIGURE 2:**
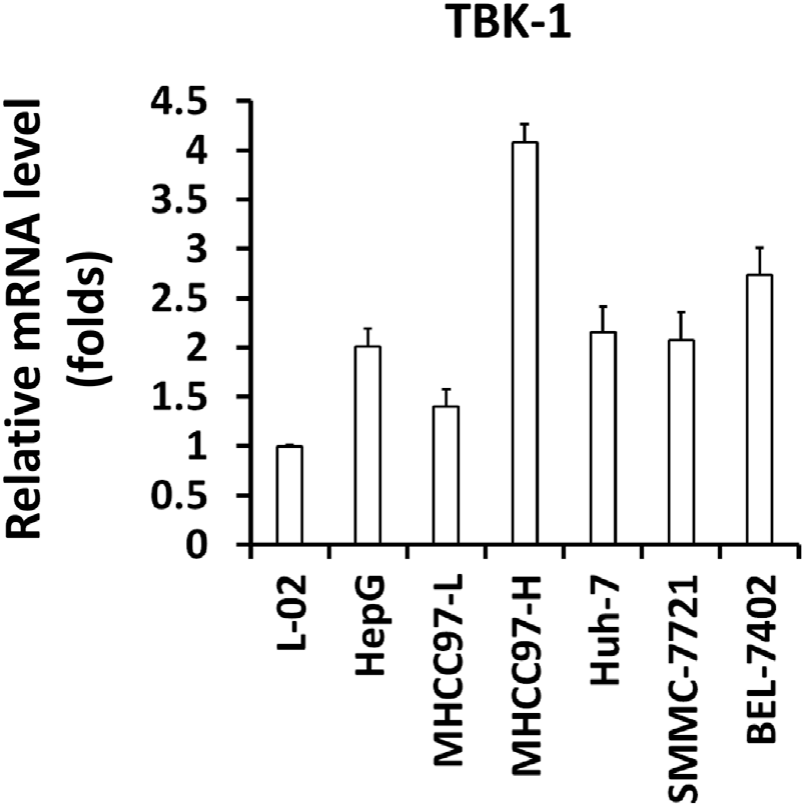
The expression level of *TBK1* in hepatic cell lines. The expression level of *TBK1* in hepatic cell lines was examined by qPCR. The results were shown as Histogram form mean ± SD.

Next, the expression of *TBK1* in hepatic cell lines was examined. As shown in Figure 2, the expression level of *TBK1* was much higher in HCC cells compared with the non-tumor hepatic cell line L-02. Among the selected HCC cells, the expression level of *TBK1* in MHCC97-H cells was the highest, while the expression level of *TBK1* in MHCC97-L cells was the lowest, and the expression level of *TBK1* in HepG2 was moderate. Because of these results, expression of *TBK1* was knocked down using siRNA in MHCC97-H cells or *TBK1* was overexpressed in MHCC97-L cells. *TBK1* was simultaneously overexpressed and knocked down in HepG2 cells.

After knockdown or overexpression of *TBK1* in HCC cells, the cells were treated with a series of doses of molecularly-targeted drugs to determine the effect of *TBK1* in HCC cell death after treatment with molecularly-targeted drugs. The results shown in Table 4, Table 5, Table 6 demonstrate that molecularly-targeted drugs can kill HCC cells in a dose-dependent manner. Overexpression of *TBK1* in MHCC97-L and HepG2 cells can significantly downregulate the killing effect of these drugs on HCC cells, and the IC_50_ values of the drugs on the cells were significantly increased (Table 4 and Table 5). Knockdown of *TBK1* with its siRNA enhanced the antitumor activation of molecular-targeted drugs on HCC cells, and the drugs’ IC_50_ values decreased (Tables 5 and Table 6). Therefore, TBK1 is associated with the resistance of HCC cells to molecular-targeted drugs and TBK1 could be considered as a promising target for HCC treatment.

**TABLE 4:** The effect of TBK1 overexpression on the sensitivity of MHCC97-L cells to molecular-target drugs, Sorafenib, Cabozentinib, Lenvatinib, Regorafenib or Anlotinib.

Drugs	Control	TBK1
	*IC*_*50*_ Values (μmol/L)	
Sorafenib	1.67 ± 0.27	8.82 ± 0.25
Cabozentinib	1.43 ± 0.32	12.89 ± 0.30
Lenvatinib	1.88 ± 0.69	9.29 ± 0.44
Regorafenib	2.13 ± 0.92	7.42 ± 1.08
Anlotinib	1.71 ± 0.36	6.13 ± 0.34

**TABLE 5:** The effect of TBK1 overexpression or knockdown on the sensitivity of HepG2 cells to molecular-target drugs, Sorafenib, Cabozentinib, Lenvatinib, Regorafenib or Anlotinib.

Drugs	Control	TBK1	siTBK1
	*IC*_*50*_ Values (μmol/L)		
Sorafenib	1.60 ± 0.20	5.22 ± 0.26	0.20 ± 0.05
Cabozentinib	1.43 ± 0.25	6.55 ± 0.52	0.27 ± 0.10
Lenvatinib	1.08 ± 0.12	4.82 ± 0.26	0.17 ± 0.06
Regorafenib	1.67 ± 0.13	5.23 ± 0.87	0.70 ± 0.01
Anlotinib	1.15 ± 0.69	3.62 ± 0.16	0.24 ± 0.15

**TABLE 6:** The effect of TBK1 knockdown on the sensitivity of MHCC97-H cells to molecular-target drugs, Sorafenib, Cabozentinib, Lenvatinib, Regorafenib or Anlotinib.

Drugs	Control	siTBK1
	*IC*_*50*_ Values (μmol/L)	
Sorafenib	0.94 ± 0.51	0.07 ± 0.00
Cabozentinib	1.24 ± 0.60	0.23 ± 0.06
Lenvatinib	0.75 ± 0.25	0.14 ± 0.04
Regorafenib	0.83 ± 0.07	0.17 ± 0.02
Anlotinib	0.56 ± 0.18	0.22 ± 0.05

### Knockdown of TANK-binding kinase 1 Repressed Drug-Resistance Related Factors

**FIGURE 3:**
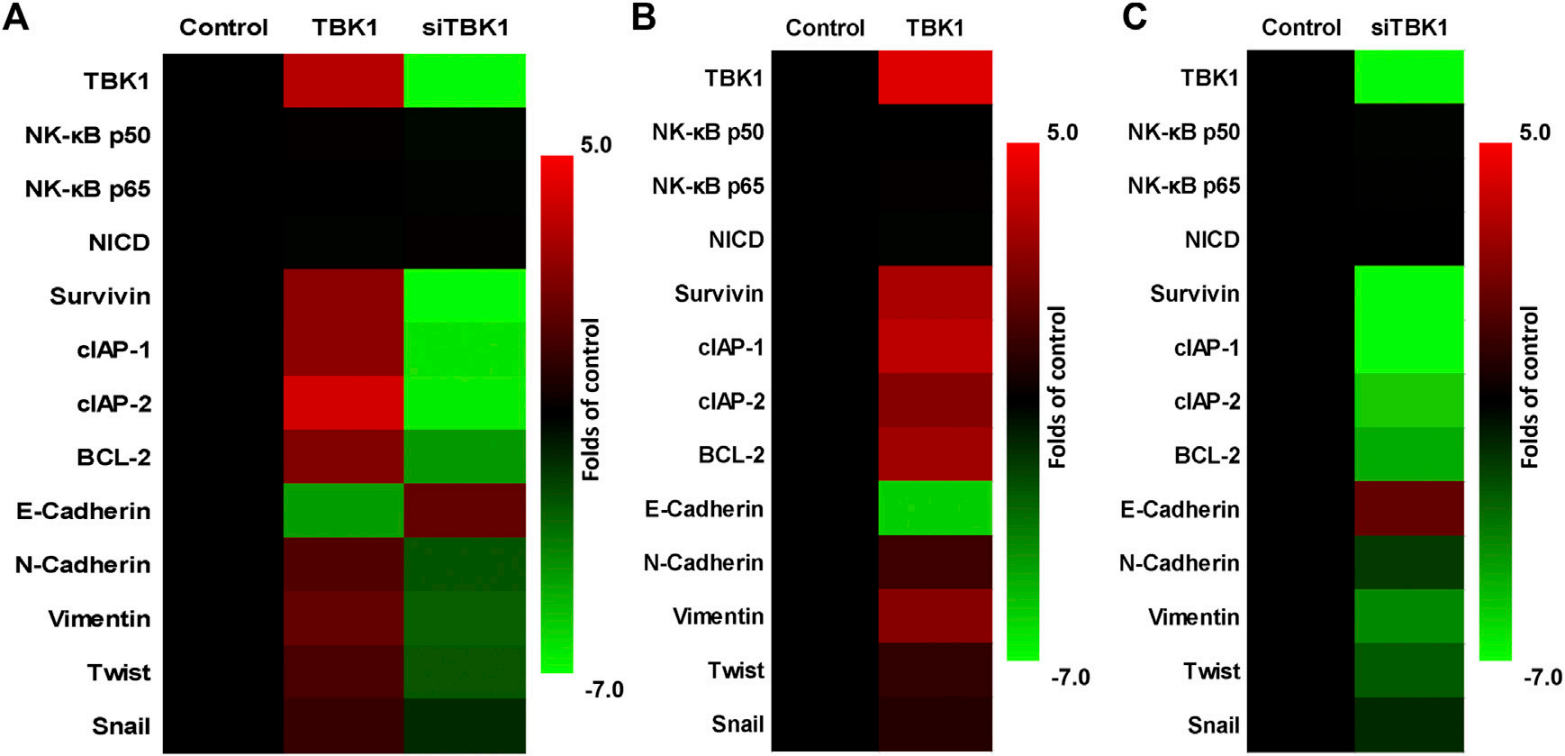
The effect of *TBK1* on the drug-resistance-related factors in HCC cells. **(A)** HepG2 cells were transfected with *TBK1* or *siTBK1* and the expression level of drug-resistance-related factors were examined by qPCR. **(B)** MHCC97-L cells were transfected with *TBK1* and the expression level of drug-resistance-related factors were examined by qPCR. **(C)** MHCC97-H cells were transfected with si*TBK1* and the expression level of drug-resistance-related factors were examined by qPCR. The results are shown as heat-map.

The effect of *TBK1* expression on drug-resistance related factors was examined by qPCR. As shown in Figure 3A, overexpression of *TBK1* in HepG2 cells upregulated drug resistance-related factors while knockdown of *TBK1* downregulated drug resistance related factors. Specifically, cellular pro-survival-/anti-apoptosis-related factors and epithelial-mesenchymal transition-related factors were affected (the expression level of E-Cadherin was upregulated or the expression level of the factors was downregulated). On this basis, TBK1 was knocked down in MHCC97-H cells, and TBK1 was overexpressed in MHCC97-L cells (Figures 3B,C). The trend of the results was basically the same as that in HepG2 (Figures 3A-C). TBK1 did not affect the expression of *NF-κB*’s *p65* or *p50*, or *Notch NICD* (FFigures 3A-C).

**FIGURE 4:**
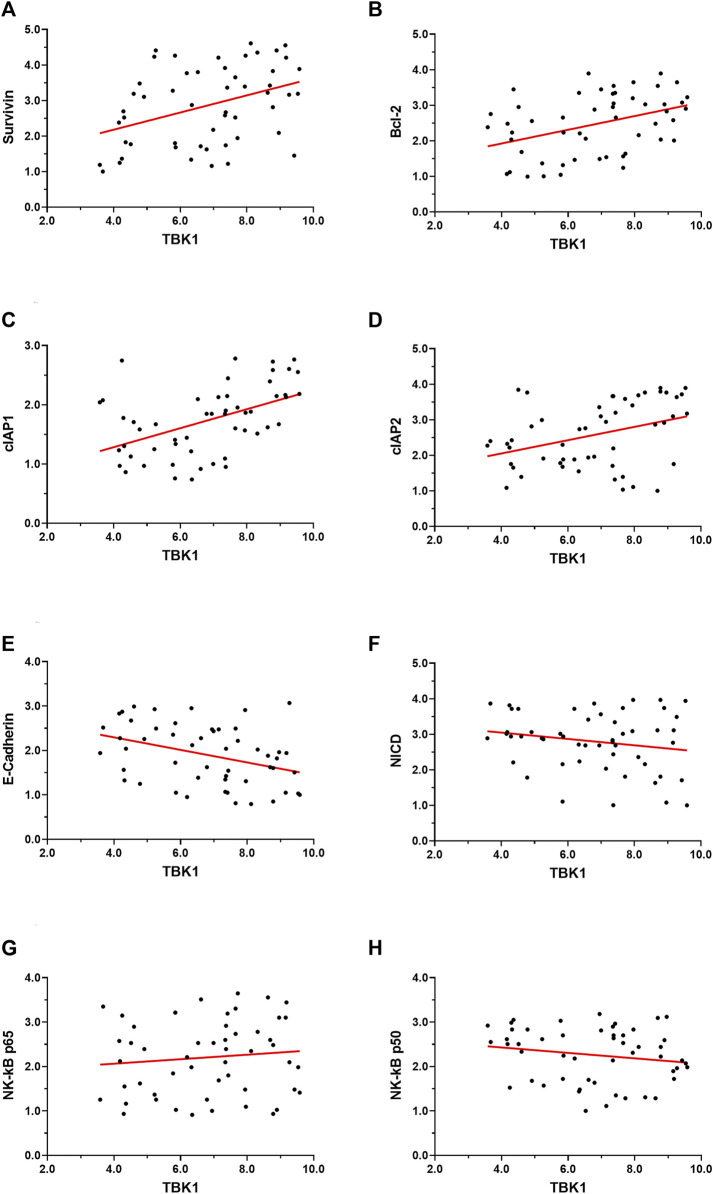
The relationship between *TBK1* with the drug-resistance-related factors in HCC specimens. The expression level of *TBK1* and drug-resistance-related factors in HCC specimens by qPCR. The expression level of *TBK1* is on the abscissa, the expression level of each factor is on the ordinate, and the data are shown as a scatter plot. Additionally, a regression equation was fit to the data (with its *p*-value) according to the trend of the scatter plot.

Next, to further confirm the effect of *TBK1*, the relationship between the expressions of *TBK1* with these factors in clinical specimens was examined (Figure 4). As shown in Figure 4, the expression level of *TBK1* was positively associated with the expression levels of *Survivin* (Figure 4A), *BCL-2* (Figure 4B), *cIAP-1* (Figure 4C), and *cIAP-2* (Figure 4D) (factors that mediate the anti-apoptosis or pro-survival of cells); negatively associated with the expression of *E-cadherin* (Figure 4E) (a typical indicator of epithelial phenotype), and did not relate to the expression of *NF-κB*’s *p65* (Figure 4F) or *p50* (Figure 4G), or *Notch NICD* (Figure 4H). Therefore, knockdown of *TBK1* repressed drug-resistance related factors’ expression level.

### Knockdown of TANK-binding kinase 1 Enhanced the *in Vivo* Sensitivity of Hepatocellular Carcinoma Cells to the Molecular-Targeted Drug Sorafenib

**FIGURE 5:**
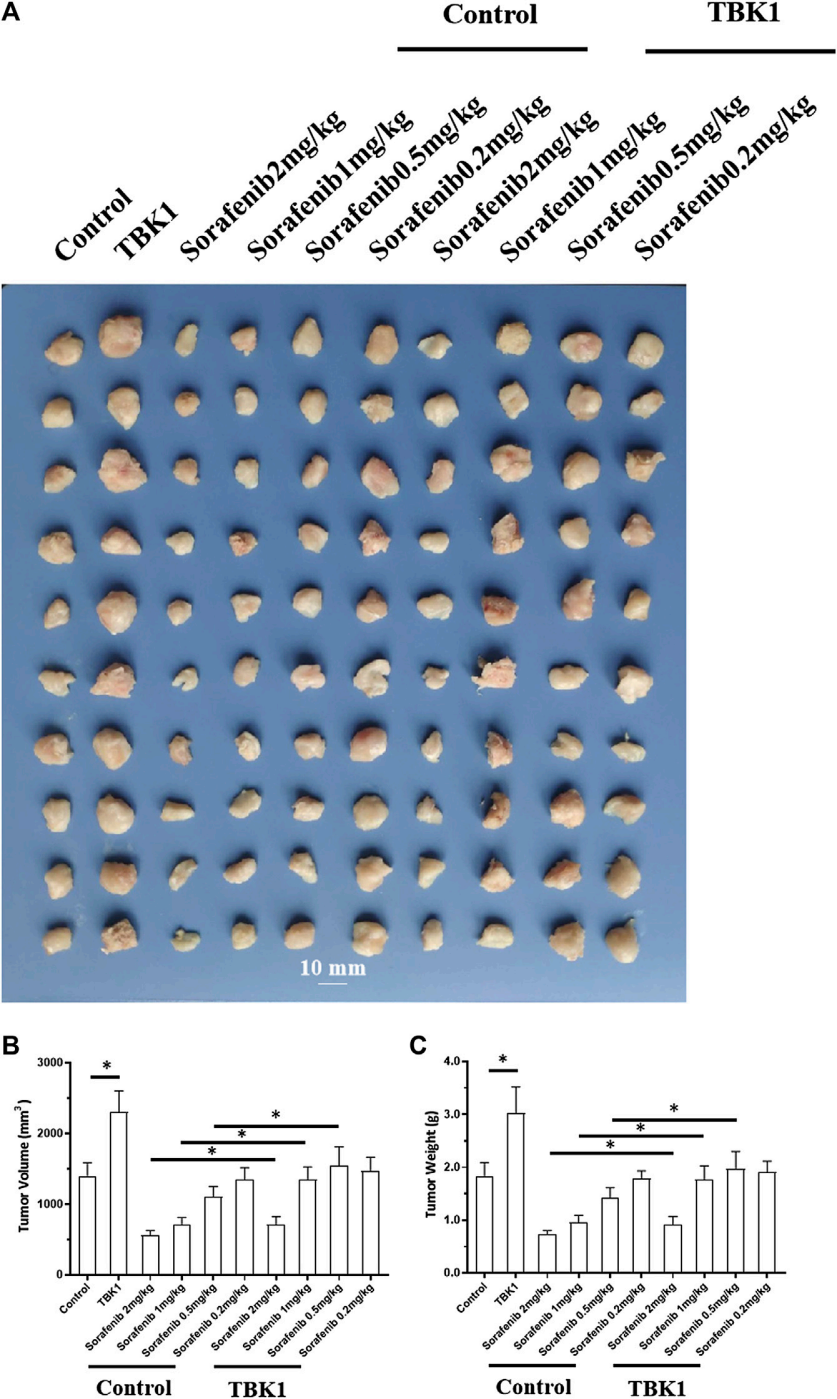
TBK1 induces resistance of MHCC97-L cells to the molecularly-targeted drug, sorafenib. After transfection of *TBK1* in MHCC97-L cells, the cells were inoculated into nude mice, and the nude mice were treated with sorafenib by oral gavage. Afterward, tumor tissues were collected to determine their volume and weight. Results are displayed as photos of tumor tissue, tumor volume, and tumor weight. *p < 0.05.

**FIGURE 6:**
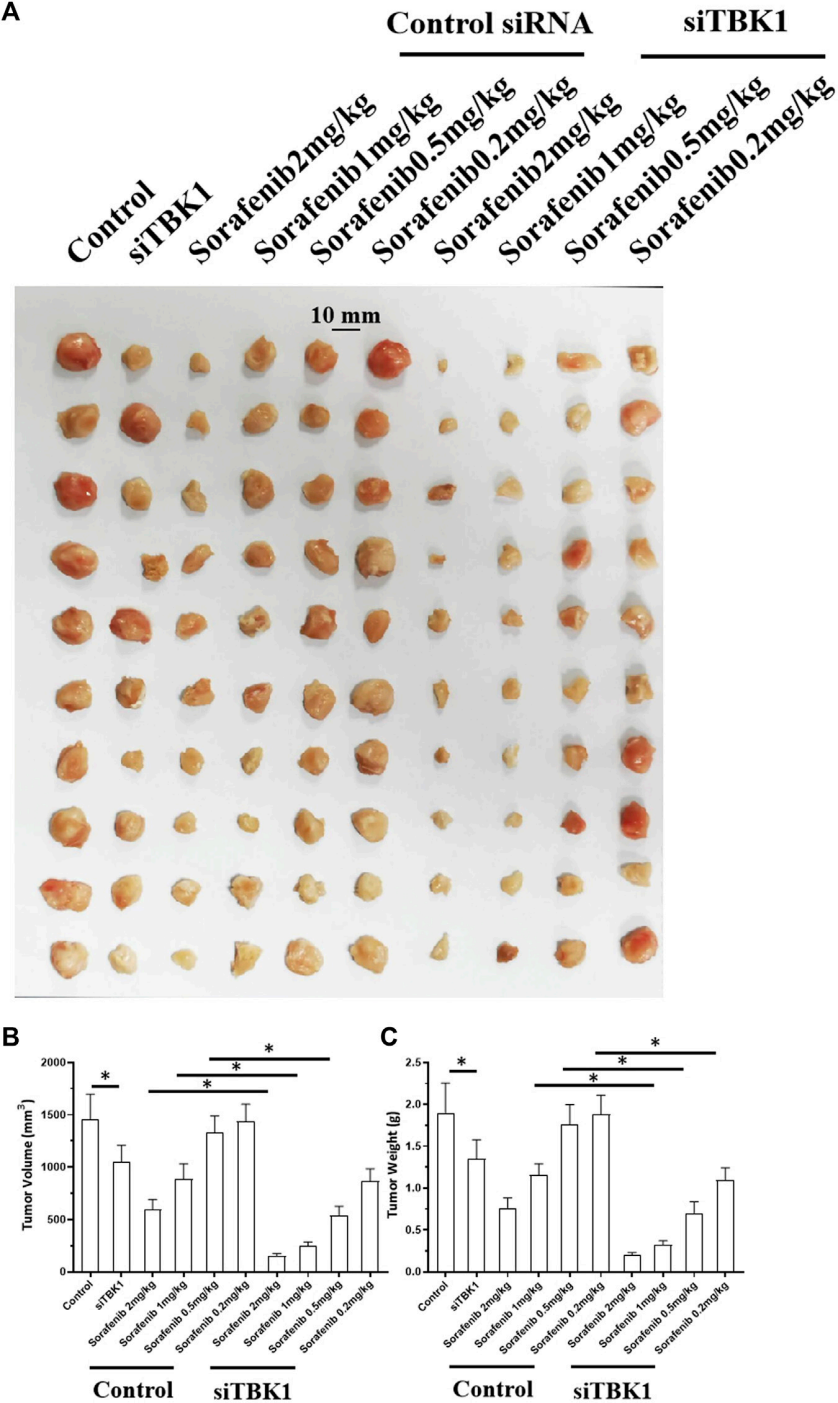
*siTBK1* enhances the sensitivity of MHCC97-H cells to the molecularly-targeted drug, sorafenib After transfection of *siTBK1* in MHCC97-H cells, the cells were inoculated into nude mice, and the nude mice were treated with sorafenib by oral gavage. Afterward, tumor tissues were collected to determine their volume and weight. Results are displayed as photos of tumor tissue, tumor volume, and tumor weight. ∗*p* < 0.05.

The above results were based on *in vitro*, cellular experiments. To further confirm the effect of *TBK1* on HCC cells, *in vivo* experiments were performed using the nude mouse model. As shown in Figures 5, 6, HCC cells (MHCC97-L and MHCC97-H) could form subcutaneous tumor tissues in nude mice. Oral administration of sorafenib inhibited the subcutaneous growth of HCC cells in a dose-dependent manner. As shown in Figure 5, overexpression of *TBK1* induced the resistance of HCC cells to sorafenib; the antitumor effect of sorafenib significantly decreased (the *IC*_*50*_ value of the indicated concentrations of sorafenib increased from 1.25 ± 0.75 mg/kg to >2 mg/kg for tumor volumes and from 1.03 ± 0.42 mg/kg to >2 mg/kg for tumor weights). Next, the results shown in Figure 6 indicate that knockdown of *TBK1 via* its siRNA in MHCC97-H cells enhanced the sensitivity of HCC cells to sorafenib; the antitumor effect of sorafenib significantly increased (the *IC*_*50*_ value of the indicated concentrations of sorafenib reduced from 1.48 ± 0.91 mg/kg to 0.32 ± 0.25 mg/kg and from 1.67 ± 0.33 mg/kg to 0.41 ± 0.10 mg/kg for tumor weights), respectively. Therefore, knockdown of *TBK1* enhanced the *in vivo* sensitivity of HCC cells to the molecular-targeted drug sorafenib.

### Knockdown of TANK-binding kinase 1 Enhanced the Sensitivity of Hepatocellular Carcinoma Cells to Cytotoxic Chemotherapeutics

The above results were all based on molecularly targeted drugs, and we further tested cytotoxic chemotherapeutic drugs to supplement them. The results are shown in Table 7. The cytotoxic chemotherapy drugs etoposide, adriamycin, paclitaxel, and gemcitabine can inhibit the survival of MHCC97-H cells in a dose–dependent manner and overexpression of TBK1 in cells can induce cell resistance to these drugs (the *IC*_*50*_ values these cytotoxic chemotherapies significantly up-regulated), and knockdown of TBK1 could significantly up-regulate the killing effects of these drugs on HepG2 cells (the *IC*_*50*_ values of these cytotoxic chemotherapeutic drugs were significantly down-regulated) (Table 7). These results further confirmed the role of TBK1 in HCC cells.

**TABLE 7:** The effect of TBK1 overexpression or knockdown in HepG2 cells to Cytotoxic chemotherapies.

Drugs	Control	TBK1	siTBK1
	*IC*_*50*_ Values (μmol/L)		
paclitaxel	0.20 ± 0.03	0.77 ± 0.24	0.04 ± 0.01
etoposide	0.62 ± 0.10	1.18 ± 0.93	0.25 ± 0.07
adriamycin	0.34 ± 0.02	1.42 ± 0.25	0.10 ± 0.09
gemcitabine	0.50 ± 0.29	1.38 ± 0.26	0.14 ± 0.05

### Small Molecular Inhibitor of TANK-binding kinase 1 Enhanced the Sensitivity of Hepatocellular Carcinoma Cells to Antitumor Drugs

**FIGURE 7:**
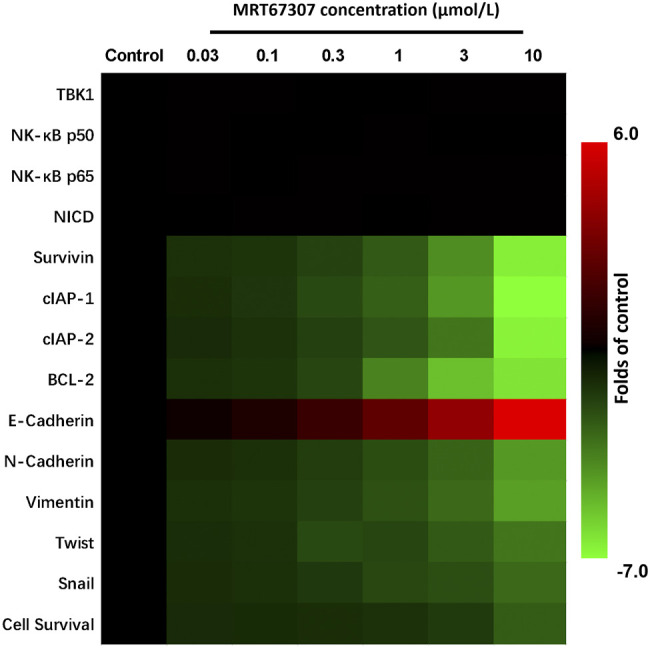
The effect of *TBK1*’s inhibitor MRT67307 on the drug-resistance-related factors in HCC cells. MHCC97-H cells were treated with the indicated concentration of MRT67307 and the expression level of drug-resistance-related factors were examined by qPCR. The inhibitory activation of MRT67307 on MHCC97-H cells was examined by MTT methods. The results are shown as heat-map.

The above results are mainly based on the use of siRNA to knock down the expression of TBK1, and further use the existing TBK1 small molecule inhibitor MRT67307 to treat HCC cells. As shown in Figure 7, MRT67307 can dose-dependently down-regulate the expression levels of NF-κB and Notch pathway-related drug resistance factors in MHCC97-H cells, and inhibited the survival of MHCC97-H cells in a dose-dependent manner. At the same time, the 1 μmol/L dose of MRT67307 itself does not have obvious cytotoxicity, but can significantly down-regulate the expression levels of NF-κB and Notch pathway-related drug resistance factors (Figure 7). Treatment of MRT67307 did not affect the expression of *NF-κB*’s *p65* or *p50*, or *Notch NICD* (Figure 7). Therefore, the 1 μmol/L dose of MRT67307 was selected for the next experiment. As shown in Table 8, treatment of 1 μmol/L dose of MRT67307 significantly up-regulate the antitumor effects of these drugs (including the molecular-targeted drugs and cytotoxic chemotherapies) on MHCC-97 cells (the *IC*_*50*_ values of these cytotoxic chemotherapeutic drugs were significantly down-regulated) (Table 8). Therefore, down-regulation of TBK1 enhanced the sensitivity of HCC cells to antitumor drugs.

**TABLE 8:** The effect of MRT67307 (1 μmol/L) on the antitumor effect of antitumor drugs in MHCC97-H cells.

Drugs	Control	MRT67307 (1 μmol/L)
	*IC*_*50*_ Values (μmol/L)	
Sorafenib	1.06 ± 0.17	0.33 ± 0.10
Cabozentinib	1.20 ± 0.39	0.20 ± 0.03
Lenvatinib	0.72 ± 0.21	0.18 ± 0.06
Regorafenib	0.95 ± 0.82	0.30 ± 0.08
Anlotinib	0.77 ± 0.43	0.14 ± 0.09
paclitaxel	0.26 ± 0.11	0.03 ± 0.00
etoposide	0.49 ± 0.12	0.10 ± 0.08
adriamycin	0.56 ± 0.18	0.15 ± 0.04
gemcitabine	0.33 ± 0.07	0.12 ± 0.07

## Discussion

At present, molecularly-targeted drug therapy for HCC is a high-interest research topic (Cerrito et al., 2021; El-Khoueiry et al., 2021; Granito et al., 2021; Vogel et al., 2021). It is generally believed that the resistance of HCC to molecularly-targeted drugs is a complex, multistep, and multifactorial process (Busche et al., 2021; Mou et al., 2021). It has been confirmed that the following factors can be involved in inducing the resistance of HCC cells to molecularly-targeted drugs: 1) mutual compensatory effect between RTKs (receptor tyrosine protein kinases), MAPK, PI3K/AKT, HGF/cMET, and other related pathways (Gao et al., 2012; Fu et al., 2020); 2) mechanisms related to cell survival and anti-apoptosis (Yang X et al., 2021; Jia et al., 2021); 3) epithelial-mesenchymal transition (Chen et al., 2021; Xia et al., 2021); and 4) many factors and mechanisms such as cancer stem cells (Ko et al., 2020; Xia et al., 2020; Leung et al., 2021). Each of these mechanism-related signaling pathways and important regulators can be used as intervention targets for HCC treatment, especially molecular-targeted-drug sensitization. There are differences in and connections between these molecular mechanisms, and knowing how to avoid inhibition of a single pathway or target and the compensatory effects of other pathways is of great importance.

TBK1 is an ideal intervention target for the sensitization of HCC to molecular-targeted drugs, mainly based on the following facts. 1) NF-κB can induce HCC by inducing the expression of survivin, cIAPs, BCL-2, and other cell pro-survival and anti-apoptotic factors; cells are resistant to molecularly-targeted drugs (Kang et al., 2013). 2) NF-κB is also regulated by Notch and other drug resistance-related pathways (Xiu et al., 2020). The Notch pathway can induce the expression of epithelial-mesenchymal transition-related factors in cells and the epithelial-mesenchymal transition phenotype through NF-κB (Kang et al., 2013; Xiu et al., 2020). 3) As a key regulator of NF-κB activation, activated TBK1 not only induces the activation of the NF-κB pathway but also induces the phosphorylation of AKT, resulting in anti-apoptotic signals and pro-cellular survival. In this study, we not only detected the effect of *TBK1* on HCC cells in molecular experiments, cellular experiments, and animal experiments but we also detected the expression of *TBK1* in clinical HCC tissue samples, confirming that TBK1 has clinical significance (Ou et al., 2011; Gao et al., 2021; Zhu et al., 2021). Theoretically, TBK1 may induce the resistance of HCC cells to molecularly-targeted drugs, and our results show that patients with a high *TBK1* expression in HCC tissues have a poor prognosis after receiving sorafenib treatment. Therefore, our results are the first to report and confirm the role of TBK1 in molecularly-targeted drug resistance in HCC.

In this study, we overexpressed and knocked down *TBK1* in HCC cells, then detected the expression levels of various factors, including: 1) survivin and cIAP related to cell survival and apoptosis -1/2, or BCL-2; 2) epithelial-mesenchymal transition-related factors, such as vimentin and N-cadherin (markers of mesenchymal transition), and E-cadherin (marker of an epithelial phenotype); 3) P65 and P50 of NF-κB; and 4) NICD of Notch protein (Zhang Y et al., 2017). These factors are all drug resistance-related genes within the NF-κB pathway and several other pathways are also involved. The results showed that *TBK1* could affect the pro-survival, anti-apoptotic, and epithelial-mesenchymal transition-related factors, but not P65, P50, or NICD. These results are also consistent with the mechanism of action of *TBK1* itself. In this study, five HCC-related, molecularly-targeted drugs were selected, but the Notch/NF-κB pathway can activate/desensitize HCC cells to molecularly-targeted drugs through cell-promoting, anti-apoptotic-related, and epithelial-mesenchymal transition-related factors, and eventually induce cell resistance to molecularly-targeted drugs. This suggests that the role of Notch/NF-κB pathway is not specific to drug selection, and downregulating *TBK1* expression is also a broad-spectrum, molecular-targeted-drug sensitization strategy in HCC. The combined effect of different drugs is of great importance. Using a variety of strategies, our research group discovered some small molecule compounds with molecularly-targeted-drug sensitization effects. Existing studies have shown that TBK1 small molecule inhibitors have certain anti-tumor activity. In the future, research on TBK1 small molecule inhibitors and the combination of TBK1 small molecule inhibitors with molecular-targeted drugs and other therapeutic strategies will be carried out. There are few reports on the role and molecular mechanism of TBK1 in HCC (only 2-3 papers in PubMed) and unclear (Kim et al., 2010; Zou et al., 2019; Jiang et al., 2021b). These articles focus on HCC-related tumor immunity and HBV-related research (Kim et al., 2010; Zou et al., 2019; Jiang et al., 2021b). This study is the first to report the relationship between TBK1 and the resistance of HCC cells to molecularly targeted drugs, which not only expands our understanding of TBK1, but also provides new ideas and implications for the treatment of HCC with molecularly targeted drugs.

In addition to the Notch/NF-κB pathway, there are other important signaling pathways in HCC cells for resistance to antitumor drugs (Zhu et al., 2019; Ma et al., 2020; He Y et al., 2021; He W et al., 2021; Guan et al., 2021; Yan et al., 2021). For example, our research group found that molecular-targeted drugs can act as ligands and agonists of the pregnane X receptor to induce the transcription factor activity and downstream drug resistance genes of PXR (Feng et al., 2018; Shao et al., 2018). These drug-resistance genes can act as enzymes of drug metabolism to accelerate clearance rate of molecularly-targeted drugs and finally induce the resistance of HCC cells to molecularly-targeted drugs. The effects of PXR and its downstream drug-resistance genes on Notch/NF-κB are similar to those of antitumor drugs, and they are all non-selective. Moreover, this study mainly focused on the role of TBK1 in HCC. In addition to HCC, TBK1 may also regulate the resistance of other malignant tumor cells to antitumor drugs (Vu and Aplin, 2014; Zhu et al., 2019; Cheng and Cashman, 2020; Zhou et al., 2020). In the future, we will further explore the impact of TBK1 and its inhibitors on other kinds of human malignancies.

## Data Availability

The original contributions presented in the study are included in the article/Supplementary Material, further inquiries can be directed to the corresponding authors.
